# Relationship Between Emotional Labor and Mental Health in Preschool Teachers: Mediation of Psychological Capital

**DOI:** 10.3389/fpsyg.2022.707961

**Published:** 2022-01-26

**Authors:** Yao Hong, Jian-Hao Huang, Jiping Zhang

**Affiliations:** ^1^The Office of Counselor, College of Teacher Education, Shaoguan University, Shaoguan, China; ^2^Department of Education Management, China-Asean International College, Dhurakij Pundit University, Bangkok, Thailand; ^3^The Office of Psychological Counselor, College of Foreign Languages, Ningbo University, Ningbo, China

**Keywords:** preschool teachers, emotional labor, mental health, psychological capital, mediation

## Abstract

This study explored the relationship among the emotional labor, psychological capital, and mental health of preschool teachers. A questionnaire survey was conducted on 411 preschool teachers in China. The results revealed the following: (1) One emotional labor strategy (Surface acting) had a significant negative effect on mental health, whereas two emotional labor strategies (expression of naturally felt emotions and deep acting) had significant positive effects. (2) The psychological capital of preschool teachers had a complete mediation on the relationship between expression of naturally felt emotions and mental health and between the deep acting and mental health.

## Introduction

Mental health is crucial to quality of teachers. Teachers’ mental health has a strong effect on students and even the entire field of education (Kovess-Masfety et al., 2007; Harding et al., 2019). Good mental health refers to an individual having no mental illness and having positive emotions, psychology, or traits, enabling the individual to feel secure in their physical, psychological, and social relationships (Diener and Seligman, 2002; Seligman, 2003; Keyes, 2005). Young children have the specific traits of cognitive infantilism, poor emotional regulation, and low socialization, which are the reasons for the particularity and high pressure of preschool teachers’ work. Teachers must meet the current urgent requirements developing of preschool education in China. Additionally, immense work pressure and challenges endangering the physical and mental health of preschool teachers affect the quality of life and level of commitment of these teachers to their work and may profoundly affect the development of young children (Buettner et al., 2016; Edward, 2016; Jeon et al., 2019). Consequently, the factors affecting preschool teachers’ mental health and the mechanisms behind these effects must be investigated to determine how the mental health of preschool teachers can be improved and thus promote the integral development of preschool education.

Many factors affect mental health of preschool teachers, and multiple studies have reported that emotional labor is a crucial factor affecting mental health (Liu et al., 2004; McLean and Connor, 2015; Katz et al., 2016). Emotional labor is the third kind of labor, other than physical labor and intellectual labor, and refers to the emotional management that an individual must perform to display an acceptable facial or physical expression in a work context to earn a reward (Hochschild, 1979; Brotheridge and Grandey, 2002; Grandey, 2003). Some studies argue that emotional labor is performed through three strategies: surface acting, which refers to only the regulation of expressions and behaviors to conform with an organization’s requirements but does not involve a change in inner feelings and is thus a disguise; the expression of naturally felt emotions, in which emotional labor is conducted automatically and emotions are revealed naturally and without the need for conscious effort; and deep acting, in which the individual manages his or her emotional state through active thinking, introduced imaginings, and memory so their internal feelings conform to the feelings expressed in the emotional expressions expected in the organization, thus producing an internally and externally consistent performance (Ashforth and Humphrey, 1993; Diefendorff et al., 2005). Researchers have demonstrated the negative effects of emotional labor at work on mental health (Kim et al., 2017; Han et al., 2018). Some studies have further shown that surface acting negatively affects mental health; greater emotional labor triggers greater work stress, which then affects mental health (Mann and Cowburn, 2005; Mesmer-Magnus et al., 2012). Philipp and Schüpbach (2010) discovered that emotional labor has a critical effect on teachers’ health, and deep acting especially benefits their mental health. Furthermore, in their study on staff in the service industry, Huang et al. (2010) discovered that surface acting negatively affected mental health whereas deep acting and expression of naturally felt emotions positively affected mental health. These studies have indicated that emotional labor affects mental health. For preschool teachers, emotional labor has the traits of long duration, high intensity, diverse emotional interactions, and painstaking effort (Yin and Lee, 2012; Zhang et al., 2020); consequently, the effect of emotional labor on the mental health of preschool teachers must be investigated to determine whether any mediators affect this relationship.

During work, employees with positive psychological capital typically have higher work positivity, which has a positive effect on their physical and mental health (Lee et al., 2016; Idris and Manganaro, 2017). Psychological capital is a type of positive psychological state experienced during growth and development; it provides an individual with emotional support and psychological energy and can weaken the adverse effects of negative emotions, positively affecting the development of the individual (Luthans et al., 2007a; Ke et al., 2009). Luthans et al. (2007b) have defined that there are four dimensions to psychological capital: self-efficacy, which is the confidence to undertake and invest the necessary effort to complete challenging tasks; optimism, which is having hope regarding current work and maintaining an attitude of positivity about future success; hope, which is the belief that success can be earned through continued hard work toward goals; and resilience, which is the character traits of perseverance and rebounding when faced with problems and adversity to achieve success. Some studies have reported that individuals with more stable psychological capital have better mental health, indicating that psychological capital has a positive effect on mental health (Psilopanagioti et al., 2012; Krasikova et al., 2015; Liu et al., 2015). Estiri et al. (2016) conducted a study investigating human resource management in hospitals and found that the psychological capital of nurses was a significant predictor of their mental health. Some studies have demonstrated that psychological capital is often a critical mediator in studies about emotional labor (Yin et al., 2018; Barbaranelli et al., 2019; Peng et al., 2019). Other studies have reported that psychological capital has a mediating effect in studies about mental health (Cole et al., 2009; Dewal and Kumar, 2017). Therefore, the present study hypothesized that psychological capital is a mediator worth considering, and in terms of emotional labor, increasing the psychological capital of preschool teachers will positively affect their mental health.

In this study, the Stress–Strain–Outcome (S-S-O) model proposed by Koeske and Koeske (1993) was employed as the theoretical basis of the study framework. The S-S-O model is used to explain the route that the stress encountered by the individual passes through to affect the individual’s psychology and behavior. Here, stress refers to a difficult and challenging objective existence; stress triggers strain in the individual, and that strain refers to the individual’s ability to instinctively judge the situation, which in turn affects the outcome. In the present study, emotional labor refers to a type of challenging labor that preschool teachers undergo while providing educational services to children, and psychological capital is the psychological resources and skills of preschool teachers instinctively responding to their work. On the basis of the S-S-O model, this study proposed that emotional labor, psychological capital, and mental health can be respectively viewed as the stress, strain, and outcome in the model; emotional labor stimulates psychological capital in preschool teachers, which indirectly affects their mental health.

In conclusion, this study investigates the relationship between emotional labor and mental health among preschool teachers and identifies the mediation of psychological capital in that relationship. This study had two major objectives: (1) to explore the relationship between emotional labor and mental health in preschool teachers and (2) to examine whether psychological capital mediates this relationship.

### Relationship Between Emotional Labor and Mental Health

Kim et al. (2017), who investigated firefighters, found that emotional labor had a significant positive effect on mental health and that mental health plans that reduce work stress and emotional labor and strengthen adaptability are critical to improving mental health. Edward et al. (2017) study of nurses found that emotional labor could inspire the personal mental health. Shu et al. (2017), in a study of nurses, discovered that the surface acting of emotional labor strategy has a significant negative effect on self-assessed anxiety in mental health, whereas deep acting had a significant positive effect on self-assessed anxiety. Researchers have identified that surface acting had a negative predictive effect on mental health of employees, whereas deep acting and expression of naturally felt emotions had positive predictive effects (Huang et al., 2010; Park et al., 2019). Summarizing these arguments, this study posited the following hypothesis:

H1: Emotional labor has a negative effect on mental health in preschool teachers.

### Relationship Between Emotional Labor and Psychological Capital

Jun (2017) found a significant correlation between emotional labor and positive psychological capital in a study of nurses. Mao and Mo (2014) discovered that for elementary school teachers, positive correlations existed between psychological capital and the emotional labor of deep acting and between psychological capital and expression of naturally felt emotions. Fu (2015) investigated preschool teachers and discovered that the emotional labor of deep acting had a significant positive effect on psychological capital. Aziz et al. (2018), in their study of professional workers, reported that surface acting was inversely correlated with psychological capital, whereas deep acting was positively correlated with psychological capital. Yin et al. (2017) investigated elementary school teachers and found that deep acting and expression of naturally felt emotions could increase the teachers’ efficacy. The following hypothesis was thus posited:

H2: Emotional labor has a positive effect on psychological capital in preschool teachers.

### Relationship Between Psychological Capital and Mental Health

Kuo et al. (2010) discovered that psychological capital was significantly correlated with mental health level, and psychological capital was a predictor of the various dimensions of mental health, such as emotional balance, internal control, and psychological conflict. Selvaraj and Bhat (2018) argued that cultivating positive psychological advantages in college students (e.g., hope, efficacy, adaptability, and optimism) can significantly raise their mental health level. Erkutlu (2014) discovered that teachers with higher psychological capital had better mental health. Van Der Meulen et al. (2018) reported that psychological capital can protect against mental health disorders to a certain extent and may even be a corrective. Taxer and Frenzel (2015) discovered that self-efficacy among teachers had a positive effect on mental health. Therefore, the following hypothesis was posited:

H3: Psychological capital has a positive effect on mental health in preschool teachers.

### Relationship Between Emotional Labor, Psychological Capital, and Mental Health

Hur et al. (2016) investigated airline service personnel and found that psychological capital played a mediating role between organizational justice and emotional labor, and positive psychological capital had significant effects on surface acting and deep acting. Yin et al. (2018) determined that among corporate employees, psychological capital had a mediating effect in the relationship between deep acting and emotional fatigue. A study on preschool teachers by Peng et al. (2019) indicated psychological capital found that psychological capital partially mediated the effects of the three types of emotional labor strategy (surface acting, expression of naturally felt emotions, and deep acting) on work burnout. Dewal and Kumar (2017) discovered that psychological capital has mediating effects on the relationship between Big Five personality traits and mental health in entrepreneurs. This literature review indicates that in preschool teachers, emotional labor affects psychological capital, and psychological capital affects mental health and may have a mediating effect between emotional labor and mental health. Therefore, the following hypothesis was posited:

H4: Psychological capital has a mediation on the relationship between emotional labor and mental health in preschool teachers.

## Methodology

### Research Model

**FIGURE 1:**
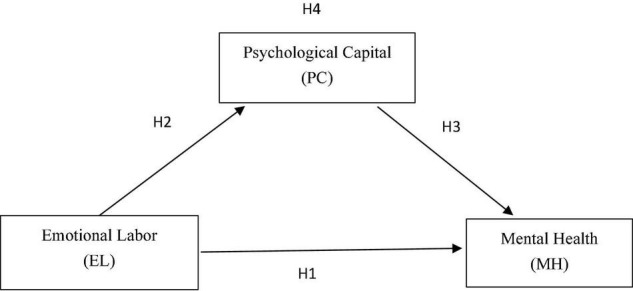
Research model. Emotional Labor (EL) includes three strategies: EL1, surface acting; EL2, expression of naturally felt emotions; EL3, deep acting.

In this study, the relationship between emotional labor and mental health in preschool teachers and the mediation of psychological capital in this relationship were investigated using emotional labor as the independent variable, mental health is the dependent variable, and psychological capital as the mediator (Figure 1).

### Sample

Tinsley and Tinsley (1987) recommend that the size of the pretest sample should be five times the number of items in the scale employed that contains the most items. The Adult mental health scale (Tseng et al., 2014) was the scale with maximum number of items that was used in this study (28 items); therefore, 158 preschool teachers were selected as the pretest sample. According to the recommendations of Gorsuch (1983), the official sample size for the study should be no fewer than five times the number of total questionnaire items; this study employed 58 items in total, and by using convenience sampling from schools willing to collaborate with the researchers, 411 preschool teachers from the Guangdong province of China were selected as the study participants. Preschool teachers in the current study referred the educators who provided enlightenment education to children aged 3–6 (Ayers, 1989; Wang et al., 2020). These teachers completed the questionnaire on wjx.cn between November 2018 and February 2019; 411 valid surveys were collected. The demographics of the respondents were as follows: 22 men (5.4%) and 398 women (94.6%); and 197 married respondents (47.9%) and 214 unmarried respondents (52.1%). In terms of education level, 97 respondents had graduated from high school or technical secondary school (23.6%), 204 had graduated from junior college (49.6%), and 110 participants had an undergraduate or higher degree (26.8%). Regarding teaching experience, 236 respondents had 5 or fewer years of teaching experience (57.4%), 50 respondents had 6–10 years of experience (12.2%), 31 respondents had 11–15 years (7.5%), 48 respondents had 16–20 years, and 46 respondents had 21 or more years of experience (11.2%). Public preschool teachers comprised 43.3% of the sample (*n* = 178), whereas private preschool teachers comprised 56.7% (*n* = 233).

### Research Instruments

#### Emotional Labor Strategy Scale

Emotional labor strategy scale which was developed by Diefendorff et al. (2005), is a 14-item self-report scale that measures three emotional labor strategies: surface acting, expression of naturally felt emotions, and deep acting. The scale was scored using a 5-point Likert scale (1 = completely disagree, 2 = disagree, 3 = neither agree nor disagree, 4 = agree, and 5 = completely agree). The sum of all item scores was the total emotional labor score; a higher score indicated a higher level of emotional labor. The Cronbach’s α, denoting internal consistency, of the pretest scale used in this study was 0.816, and items with factor loading lower than 0.40 were eliminated through exploratory factor analysis. One item was eliminated, leaving 13 items. The fit indexes for the confirmatory factor analysis results for the formal scale were as follows: χ^2^/df = 4.659, GFI = 0.904, NFI = 0.844, IFI = 0.873, NNFI = 0.839, CFI = 0.872, and RMSEA = 0.940. Therefore, the model fit met or approached the standard (Hair et al., 1998).

#### PsyCap Questionnaire

This study used the PsyCap questionnaire (PCQ-24) of Luthans et al. (2007a). The 24 items in the questionnaire assess self-efficacy, hope, optimism, and resiliency dimensions. The scale was scored using the same 5-point Likert scale used for the Emotional Labor Scale. The sum of all item scores was the total psychological capital score, and a higher score indicated a higher level of psychological capital. The Cronbach’s α of the pretest questionnaire used in this study was 0.967, and items with factor loading lower than 0.40 were eliminated through exploratory factor analysis. Six items were eliminated, leaving 18 items. The fitting indexes for the confirmatory factor analysis results were as follows: χ^2^/df = 2.618, GFI = 0.907, NFI = 0.935, IFI = 0.959, NNFI = 0.951, CFI = 0.959, and RMSEA = 0.063. Therefore, the model fit met the standard (Hair et al., 1998).

#### Adult Mental Health Scale

This study employed the Adult mental health scale designed by Tseng et al. (2014), which comprises 28 items covering five dimensions—somatic symptoms, anxiety, social undermining, depression, and positive mentality. For consistent scoring, positive mentality was changed to negative mentality. The scale was scored using the same 5-point Likert scale as for the other two measures. The sum of all item reverse scores was the total mental health score, and a higher score indicated a higher level of mental health. The Cronbach’s α of the pretest scale used in this study was 0.959, and items with factor loading lower than 0.40 were eliminated through exploratory factor analysis. One item was eliminated, leaving 27 items. The fitting indexes for the confirmatory factor analysis results were as follows: χ^2^/df = 2.338, GFI = 0.883, NFI = 0.904, IFI = 0.943, NNFI = 0.936, CFI = 0.943, and RMSEA = 0.057. Therefore, the model fit met the standard (Hair et al., 1998).

### Statistical Analysis Method

Data were analyzed using the package SPSS and AMOS. Descriptive statistics, Cronbach’s α, and Pearson correlation were performed using SPSS. Confirmatory factor analysis, structural equation modelling (SEM) and bootstrapping were performed using AMOS.

## Results

### Common Method Deviation Control and Verification

In this study, data were collected through a questionnaire; common method bias may thus have occurred. To test for common method bias, strict program controls were adopted during the questionnaire process, and it was emphasized that the survey results would be used only for academic research and the survey responses would be anonymous and confidential. Furthermore, Harman’s single-factor test was used in the data analysis process to test for common method bias (Harman, 1967). Factor analysis of unrotated factors revealed that the first factor explained only 29.889% of the variance, which was lower than the 40% critical standard value; therefore, this study did not have any severe common method biases.

### Descriptive Statistics and Correlation Analysis for Each Variable

Ranked in order from highest to lowest score, the strategies of emotional labor in preschool teachers were expression of naturally felt emotions [mean (M) = 3.654, standard deviation (SD) = 0.679], deep acting (M = 3.604, SD = 0.767), and surface acting (M = 2.692, SD = 0.785); this revealed that the preschool teachers more often used expression of naturally felt emotions and deep acting in their emotional labor at work, using surface acting less often. The preschool teachers’ average psychological capital (M = 3.711, SD = 0.574) was higher than the median score (3), indicating that the preschool teachers had above-average psychological capital. Their average mental health score (M = 3.782, SD = 0.660) was also higher than the median value (3), demonstrating that the preschool teachers had above-average mental health.

See Table 1. A significant negative correlation was discovered between surface acting and mental health (*r* = –0.123, *p* < 0.05), a significant positive correlation between deep acting and mental health (*r* = 0.186, *p* < 0.01), and a significant positive correlation between expression of naturally felt emotions and mental health (*r* = 0.225, *p* < 0.01). Surface acting was not significantly correlated with psychological capital (*r* = 0.052, *p* > 0.05), deep acting was significantly positively correlated with psychological capital (*r* = 0.497, *p* < 0.01). Expression of naturally felt emotions was significantly positively correlated with psychological capital (*r* = 0.545, *p* < 0.01). Psychological capital and mental health were significantly positively correlated (*r* = 0.431, *p* < 0.01). Most of the variables considered in this study exhibited significant correlations, and the correlation coefficients were all in the range –0.123 to 0.545, which did not indicate collinearity problems. Regression analysis was thus applicable.

**TABLE 1:** Descriptive statistics and correlation analysis.

Variable	*M*	SD	EL1	EL2	EL3	PC	MH
EL1	2.692	0.785	1				
EL2	3.654	0.679	0.014	1			
EL3	3.604	0.767	0.348**	0.414**	1		
PC	3.711	0.574	0.052	0.545**	0.497**	1	
MH	3.782	0.659	–0.123*	0.225**	0.186**	0.431**	1

*n = 411; EL1, surface acting; EL2, expression of naturally felt emotions; EL3, deep acting; PC, psychological capital; MH, mental health.*

*p < 0.05.

**p < 0.01.

***p < 0.001.

### Structural Model

Hayes and Rockwood (2017) revealed that bootstrapping is an effective and accurate mediation test method. Therefore, SEM and bootstrapping were the primary statistical analysis methods to verify the mediation in the current study. The adoption of SEM suggests using three or more observed variables for each latent variable (Russell et al., 1998). Taking psychological capital as an example, the questionnaire included four dimensions: self-efficacy, hope, optimism, and resiliency. The current study adopted these four dimensions as observed variables of latent variables. Considering mental health as an example, the scale included five dimensions: somatic symptoms, anxiety, social undermining, depression, and positive mentality, and the current study adopted these five dimensions as observed variables of latent variables. However, the emotional labor strategy scale was divided into three different strategies: surface acting, expression of naturally felt emotions, and deep acting. Therefore, emotional labor strategy cannot be aggregated into one latent variable and must be regarded as three latent variables. The current study adopted items as the observed variables of these three latent variables. In the current study, the thresholds set for a good model fit as follows: χ^2^/df < 5 (Schumacker and Lomax, 2012), GFI ≧ 0.800, IFI ≧ 0.800, TLI ≧ 0.800, CFI ≧ 0.800 (Gefen et al., 2000), RMSEA ≦ 0.100 (Browne and Cudeck, 1993).

The current study evaluated structural models in three steps. In the first step, we observed at the direct association between emotional labor and mental health. We discovered a significant negative association between surface acting and mental health (β = –0.201, *p* < 0.010), a significant positive correlation between expression of naturally felt emotions and mental health (β = 0.123, *p* < 0.050), and deep acting and mental health (β = 0.133, *p* < 0.05). The *R*^2^ value for this model was 0.146. The fit indices—χ^2^/df = 3.781, GFI = 0.885, IFI = 0.879, TLI = 0.856, CFI = 0.878, and RMSEA = 0.082—revealed that a satisfactory model fit was achieved. Thus, H1 was supported partially.

**FIGURE 2:**
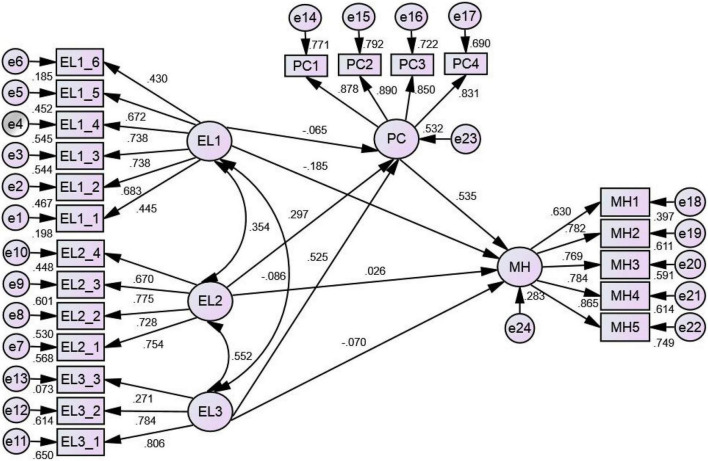
Structural model—Mediational model. EL1, surface acting; EL2, expression of naturally felt emotions; EL3, deep acting; PC, psychological capital; MH, mental health.

In the second step, we established a structural model—mediational model (Figure 2), in which psychological capital was introduced as the mediator of the relationship between emotional labor and mental health. The fit indices—χ^2^/df = 3.315, GFI = 0.871, IFI = 0.899, TLI = 0.882, CFI = 0.898, and RMSEA = 0.075—revealed that the structural model exhibited a satisfactory fit, suggesting that the psychological capital as a mediator of the relationship between emotional labor and mental health was crucial.

Finally, we performed bootstrapping by specifying a sample of size 2,000 in AMOS to examine the significance of the mediator. The results of the mediational model (Table 2) revealed that after the inclusion of the mediator, the indirect effects of the relationship between surface acting (EL1) and mental health (β = –0.035, and 95% confidence interval did overlap with zero) exerted no mediation. However, the indirect effects of the relationship between expression of naturally felt emotions (EL2) and mental health (β = –0.159, and 95% confidence interval did no overlap with zero) as well as deep acting (EL3) and mental health (β = 0.281, and 95% confidence interval did not overlap with zero) indicated a significant mediation. Furthermore, the direct effects of the relationship between expression of naturally felt emotions (EL2) and mental health (β = 0.026, and 95% confidence interval did overlap with zero) as well as deep acting (EL3) and mental health (β = –0.070, and 95% confidence interval did overlap with zero). Therefore, psychological capital exhibited a complete mediation on the relationship between expression of naturally felt emotions (EL2) and mental health and deep acting (EL3) and mental health.

**TABLE 2:** Direct and indirect effects and 95% confidence intervals.

Path relationship	Estimate	95% Lower	95% Upper
**Standardized direct effects**			
EL1 → PC	–0.065	–0.192	0.047
EL2 → PC	0.297	0.114	0.461
EL3 → PC	0.525	0.346	0.690
PC → MH	0.535	0.370	0.710
EL1 → MH	–0.185	–0.321	–0.047
EL2 → MH	0.026	–0.142	0.189
EL3 → MH	–0.070	–0.270	0.128
**Standardized Indirect Effects**			
EL1 → PC→ MH	–0.035	–0.111	0.024
EL2 → PC→ MH	0.159	0.067	0.265
EL3 → PC→ MH	0.281	0.165	0.456

*Empirical 95% confidence interval does not overlap with zero indicates significant statistically; n = 411 (bootstrapping by specifying a sample of size 2,000); EL1, surface acting; EL2, expression of naturally felt emotions; EL3, deep acting; PC, psychological capital; MH, mental health.*

## Conclusion and Discussion

### Among Preschool Teachers, Surface Acting Had Significant Negative Effect on Mental Health, Whereas Deep Acting and Expression of Naturally Felt Emotions Had Significant Positive Effect

The results of this study revealed that among preschool teachers, surface acting lowers mental health, whereas deep acting and expression of naturally felt emotions improve mental health. This is consistent with the findings of Huang et al. (2010) and Philipp and Schüpbach (2010). If preschool teachers adopt surface acting, they disguise and control their actual emotions to different degrees so they can display the emotions required in the kindergarten setting. Especially when teachers need to express negative emotions in order to express emotions not consistent with their actual emotions, surface acting for long periods of time triggers psychological discomfort and leads to mental health problems. Preschool teachers who express their naturally felt emotions have a more natural and sincere experience and express the emotions needed in a preschool environment; this strategy of emotional work enables preschool teachers to maintain a better mental state and has a positive effect on mental health. Preschool teachers using deep acting express emotions that are internally and cognitively consistent with the emotional expressions required by the environment of kindergarten; this helps maintain the teachers’ passion for their careers and improves their mental health.

### Expression of Naturally Felt Emotions and Deep Acting Have Significant Positive Effect on Psychological Capital in Preschool Teachers

The study findings demonstrated that expression of naturally felt emotions and deep acting increase psychological capital in preschool teachers. This is consistent with the findings of Fu (2015) and Aziz et al. (2018). Preschool teachers who use the expression of naturally felt emotions strategy do not need to disguise or suppress their emotions and do not expend too much physical or mental energy, which has a positive effect on psychological capital. It would be preschool teachers express emotions such as enthusiasm, optimism, and kindness naturally when facing lovely children, which strengthens the psychological capital of preschool teachers. In addition, Those using deep acting must expend more effort on emotion and mental energy regulation, but deep acting increases inner harmony, which improves work performance and increases psychological capital. The possible reason is that preschool teachers were usually able to adjust internal cognition, change emotional expression based on the noble professional image and professional ethics of teachers, then strengthen the psychological capital of preschool teachers.

### Psychological Capital Complete Mediating the Relationship Between Expression of Naturally Felt Emotions and Mental Health and Between Deep Acting and Mental Health

The study findings further revealed the mechanism through which emotional labor affects mental health. Expression of naturally felt emotions and deep acting were discovered to affect mental health through psychological capital. Accordingly, Preschool teachers would adopt two strategies of emotional labor to affect mental health through the complete mediation of psychological capital. Firstly, preschool teachers adopt expression of naturally felt emotions to express true emotional state exactly with the emotional requirement of kindergarten. The work expression of revealed naturally would strengthen the psychological capital of preschool teachers and improve their mental health. Secondly, preschool teachers adopt deep acting to manage internal emotional states through active thinking, introducing imagination, memory, so that their inner feelings are accord with the requirement of kindergarten to achieve a emotional consistent of internal and external, would also strengthen the psychological capital of preschool teachers, which improves their mental health. This is resemblance to the findings of past empirical studies, verified that psychological capital plays a crucial and vital mediating role on the relationship between related variables of emotional labor and mental health (Dewal and Kumar, 2017; Yin et al., 2018; Peng et al., 2019).

This is in agreement with the S-S-O model of Koeske and Koeske (1993). The S-S-O model associates stress with outcome. Stress is generated by environmental demands that affect individuals. The unbalanced feelings between personal motivation and environmental demands that is perceived as irritating, troublesome or destructive. Preschool teachers might have unbalanced feelings due to the imbalance between personal responsibility or mission and the requirements of kindergarten managers or expectations of child’s parents. Therefore, emotional labor in this study can be regarded as a kind of stress. As advocated by the S-S-O model, stress causes strain, which affects the individual emotional and psychological response. Emotional labor of preschool teachers triggers a positive mental state to provide emotional support and mental energy such as optimism, resilience, hope. Therefore, psychological capital can be regarded as a kind of strain to improve the mental health of preschool teachers. In preschool education, either of deep acting or expression of naturally felt emotions affects teachers’ consumption of psychological resources and affects mental health through changes in psychological capital. In consequence, the study proposes a research framework with psychological capital as mediation to explore the relationship between emotional labor and mental health of preschool teachers. In addition to extending the application of the S-S-O model in empirical research, it also enhances the richness of preschool education practice.

## Suggestions

### Encourage Preschool Teachers to Adopt Appropriate Emotional Labor Strategies

This study discovered that in preschool teachers, surface acting has a negative predictive effect on mental health, whereas deep acting and expression of naturally felt emotions have positive predictive effects. Therefore, this study presented the following suggestions for Kindergartens and Education sector:

Education sector is recommended to include a course on emotional management and mental relief as mandatory classes in the 18 h of professional training for preschool teachers, this course could improve preschool teachers’ emotional self-awareness and self-management skills and help them acquire emotional labor strategies. They could be taught to avoid surface acting at work and instead use deep acting and expression of naturally felt emotions.

Kindergartens should organize group activities of quality development actively to improve the expression of deep acting and expression of naturally felt emotions, such as enthusiasm, optimism, love, trust, kindness, and friendliness. Through group activities, create a working atmosphere of mutual help, improve the sense of belonging of preschool teachers, and thus love preschool education and the kindergarten where they are located.

Preschool teachers should pay attention to their individual growth and career development, tapping into their inner sense of mission and potential and adjusting their emotional labor. These measures would promote mental health, enabling preschool teachers to confront their work and life with a positive, sunny attitude.

### Reinforce Psychological Capital Training for Preschool Teachers

In this study, preschool teachers’ psychological capital was found to have complete mediation on the relationship between deep acting and mental health and that between expression of naturally felt emotions and mental health. Therefore, this study presented the following suggestions:

Government departments for education should formulate preschool education policies that increase psychological capital for preschool teachers, reinforce the self-efficacy, hope, optimism, and resiliency of preschool teachers through the reward design process, organization of training programs with manager awareness.

Kindergartens should implement psychological capital training of teachers to optimize their mental health, such as organizing group-style assistance and intervention in psychological capital activities. Kindergartens should provide opportunities for preschool teachers to participate in management decision-making and improve their sense of accomplishment.

Preschool teachers should attend community, kindergarten teacher exchanges and family activities to expand their social exchange, and reasonable planning of life, leisure and sports time, increasing their psychological capital and bolstering their mental health.

## Research Limitations and Future Directions

This study only obtained 411 valid questionnaires from Guangdong province of China for analysis that suggested future researchers can expand the sample size or analyze and compare different regions to understand the differences. The effect of emotional labor on preschool teachers’ mental health was investigated through a cross-sectional study. In the future, researchers can conduct a longitudinal study to conduct more in-depth investigations. The method of data collection in this study was a questionnaire, and researchers could instead use case studies or qualitative research methods to more broadly discuss the variables considered in this study and expand on the study’s findings. On the basis of this study, a moderated mediation model can be considered by adding other relevant variables such as workplace social support (Ju et al., 2015), or school climate (McLean et al., 2017). More rigorously, future research can consider adding relevant contextual variables for discussion, such as urban/rural or kindergarten size, etc., to deepen the analysis of research results.

## Data Availability Statement

The original contributions presented in the study are included in the article/supplementary material, further inquiries can be directed to the corresponding author.

## Author Contributions

YH: conceptualization, methodology, investigation, formal analysis, writing—original draft, and visualization. J-HH: supervision. J-HH and JZ: writing—review and editing. All authors contributed to the article and approved the submitted version.

## Conflict of Interest

The authors declare that the research was conducted in the absence of any commercial or financial relationships that could be construed as a potential conflict of interest.

## Publisher’s Note

All claims expressed in this article are solely those of the authors and do not necessarily represent those of their affiliated organizations, or those of the publisher, the editors and the reviewers. Any product that may be evaluated in this article, or claim that may be made by its manufacturer, is not guaranteed or endorsed by the publisher.
